# Invasive group A *Streptococcus* disease in Australian children: 2016 to 2018 – a descriptive cohort study

**DOI:** 10.1186/s12889-019-8085-2

**Published:** 2019-12-30

**Authors:** Jane Oliver, Elise Thielemans, Alissa McMinn, Ciara Baker, Philip N. Britton, Julia E. Clark, Helen S. Marshall, Christopher C. Blyth, Joshua Francis, Jim Buttery, Andrew C. Steer, Nigel W. Crawford, on behalf of the PAEDS investigators, R. Booy, J. Connell, R. Dale, M. Deverell, N. Dinsmore, S. Dougherty, C. Finucane, M. Gibson, M. Gold, C. Heath, L. Hickie, T. Hutchinson, C. Jones, J. Jones, J. Kent, H. Knight, A. Kynaston, D. Lee, G. Lewis, S. Low, N. Maclean, K. Macartney, F. McDonald, N. McLaren, J. McRae, J. Murphy, K. Meredith, M. Nissen, C. Orr, K. Orr, N. Phillips, M. Pym, J. Quinn, P. Richmond, L. Rhind, A. Roberts, C. Robins, L. Rost, J. Royle, G. Saravanos, T. Snelling, C. Talbott, S. Tan, L. Trinh, L. Vidler, M. Walker, R. West, C. Wharton, N. Wood, Y. Zurynski

**Affiliations:** 1 Murdoch Children’s Research Institute, Royal Children’s Hospital, Flemington Rd, Parkville, Victoria 3052 Australia; 2 The Peter Doherty Institute for Infection and Immunity, University of Melbourne, Melbourne, Victoria Australia; 3 Université Libre de Bruxelles, Bruxelles, Belgium; 4 The Children’s Hospital at Westmead, Sydney, Australia; 5 Medical School University of Sydney, Sydney, New South Wales Australia; 6 Queensland Children’s Hospital, and School of Clinical Medicine, University of Queensland, Brisbane, Queensland Australia; 7 Women’s and Children’s Hospital, Adelaide, South Australia Australia; 8 School of Medicine angeid Telethon Kids Institute, University of Western Australia, Perth, Australia; 9 Perth Children’s Hospital, Perth, Western Australia Australia; 10 PathWest Laboratory Medicine, Nedlands, Perth, Australia; 11 Royal Darwin Hospital, Darwin, Northern Territory Australia; 12 Menzies School of Health Research, Darwin, Northern Territory Australia; 13 Monash Health, Monash University, Melbourne, Victoria Australia

**Keywords:** Group A Streptococcus, Child health, Infectious diseases, Public health, Invasive

## Abstract

**Objectives:**

Invasive group A *Streptococcus* (iGAS) disease is serious and sometimes life-threatening. The Paediatric Active Enhanced Disease Surveillance (PAEDS) Network collects voluntary notifications from seven major Australian paediatric hospitals on patients with certain conditions, including iGAS disease. Our aims were to: 1) Describe the epidemiological distribution of paediatric iGAS disease in Australia and correlate this with influenza notifications, 2) Identify GAS strains commonly associated with invasive disease in children.

**Methods:**

IGAS and influenza notification data were obtained (from the PAEDS Network and the Australian Institute of Health and Welfare, respectively, for the period 1 July 2016 to 30 June 2018). Included iGAS patients had GAS isolated from a normally sterile body site. Data were described according to selected clinical and demographic characteristics, including by age group and Australian State, with proportions and minimum incidence rates estimated.

**Results:**

A total of 181 patients were identified, with most (115, 63.5%) <5 years old. The mean annual minimum incidence rate was 1.6 (95% confidence interval: 1.1–2.3) per 100,000 children across the study period. An epidemiological correlation with the seasonal burden of influenza was noted. Contact prophylaxis was not consistently offered. Of 96 patients with *emm-*typing results available, 72.9% showed *emm*-1, −4 or − 12.

**Conclusions:**

Robust surveillance systems and cohesive patient management guidelines are needed. Making iGAS disease nationally notifiable would help facilitate this. Influenza vaccination may contribute to reducing seasonal increases in iGAS incidence. The burden of disease emphasises the need for ongoing progress in GAS vaccine development.

## Background

Group A *Streptococcus* (GAS) produces a wide range of illnesses in humans. Invasive GAS (iGAS) diseases are associated with acute mortality and considerable morbidity, including permanent disability. IGAS disease is defined by the isolation of GAS from a normally sterile bodily site [1]. Examples of iGAS disease include: septic arthritis, osteomyelitis, meningitis, bacteremia/septiceamia, pneumonia, and necrotizing fasciitis. Even in high-income countries, these conditions are often associated with case fatality rates (CFR) of approximately 10–15%; with the CRF higher still for necrotizing fasciitis (CFR approximately 20%) and streptococcal toxic shock syndrome (STSS; recent CFRs reported as ≤28%) [2–5]. A global 2005 review estimated that over 660,000 people develop iGAS disease worldwide each year, with over 160,000 resulting deaths [6]. Much of the disease burden is concentrated in low/middle-income countries [6]. People with the highest risk of iGAS disease include young children, elderly people, injecting drug users, and patients with certain comorbidities such as diabetes, influenza and immunosuppression. Patients’ close contacts have an approximately 2000-times increased risk of developing iGAS disease themselves; termed ‘secondary disease’ [7, 8]. This increased risk is especially pronounced for mother-neonate pairs and co-habiting couples aged > 74 years old [8]. Despite this, there are no official Australian guidelines concerning the prevention of secondary disease using contact prophylaxis. Disparate clinical guidelines from other high-income countries and some Australian States (Victoria, Queensland, New South Wales, Northern Territory) exist [1, 9–15].

The incidence of iGAS disease has increased across several high-income regions for unclear reasons, with 2017/2018 rates of 7–10 per 100,000 across the general population reported in the United States (US) and Canada [16–20]. One possible explanation may involve the increasing diversity in *emm-*types [20, 21]. Socioeconomic drivers may have an important role in promoting iGAS disease, particularly excessive drug and alcohol use [20, 22]. The incidence of iGAS disease among First Nations peoples of Canada exceeded 30/100,000 during 2009–2014, [23, 24] which was comparable to rates observed for Indigenous populations in Australia and New Zealand [25–28]. Inequitably high rates of iGAS disease have also been documented among US First Nations peoples [29]. A study set in the Northern Territory of Australia study estimated an iGAS incidence rate of 70/100,000 for the Indigenous population as a whole, almost 8-fold higher than that reported for the non-Indigenous population [25]. An Australian study in Queensland noted high ethnic inequities amongst children aged < 19 years-old, with an annualised incidence of 13.2/100,000 for Indigenous children, nearly 4-fold higher than for non-Indigenous children [26].

Local Australian surveillance data indicates that the incidence rate of iGAS disease is increasing in Victoria, with a new high level reached in 2017, of 3.6/100,000 (95% confidence interval, CI: 3.2–4.1/100,000; i.e. 220 new cases that year) [30, 31]. A 2017 outbreak among Victorian children is especially concerning, and was associated with a high burden of seasonal influenza [31]. As iGAS disease is currently notifiable only in two Australian jurisdictions (Northern Territory & Queensland), it is difficult to accurately ascertain the burden of disease [10, 12]. During 2017–2018, 143 patients were notified in the Northern Territory [32]. In Queensland, a new high number of 381 patients were notified in 2017 (and 355 patients were notified in 2018). Of the 2017–2018 Queensland total, 16.2% of patients were aged <20 years [33].

The Paediatric Active Enhanced Disease Surveillance (PAEDS) Network compiles detailed information on selected serious childhood conditions treated in seven major paediatric hospitals across Australia [34]. IGAS disease was included as a condition of interest in July 2016, however not all states commenced data collection at the same time and retrospective recruitment was frequent (Additional file 2: Figure S1). This national surveillance followed a pilot study at one of two participating PAEDS sites in Melbourne, Victoria over 2014–2016 (the Royal Children’s Hospital, RCH) [30]. A key goal of the PAEDS Network was to enhance the understanding the disease burden and provide data to support targeted control and prevention activities, including vaccination [34]. The major limitation of the PAEDS Network is that data collected are limited to patients treated in participating hospitals. Patients treated at non-notifying hospitals are missed by this system.

Currently there is no vaccine to prevent iGAS disease. However, vaccine development is underway in the US, Brazil, Europe and in Australia, with endorsement from the World Health Organization (WHO). Important steps in vaccine development include establishing the burden of disease and associated financial costs, as well as identifying GAS strains causing invasive disease [35–37].

Accordingly, this research utilises PAEDS Network data with aims to: 1) Describe the epidemiological distribution of paediatric iGAS disease and correlate this with the seasonal burden of influenza, and 2) Identify any GAS strains that are particularly likely to be associated with invasive disease in children.

## Methods

Surveillance of iGAS disease was conducted over 24 months from July 1, 2016 to June 30, 2018 at seven major paediatric centres in Australia; Royal Children’s Hospital (RCH) and Monash Children’s Hospital (MCH; Victoria), Queensland Children’s Hospital (QCH; Queensland), Children’s Hospital at Westmead (CHW; New South Wales), Royal Darwin Hospital (RDH; Northern Territory), Perth Children’s Hospital (PCH; Western Australia), Women’s and Children’s Hospital (WCH; South Australia; Additional file 3: Figure S2). Children with iGAS disease who were seen in settings other that those hospitals listed above were not able to be included in this analysis.

### Data sources

Surveillance data were collected under the auspices of the national PAEDS Network. (http://www.paeds.edu.au/) [34]. RCH and MCH conducted prospective recruitment throughout the entire 24 month study period. The other five centres initiated prospective surveillance at various times during the study period, with retrospective recruitment to detect patients diagnosed earlier during the study period (the prospective surveillance period ranged from 13 to 20 months) because of varied timing for ethics approvals (Additional file 2: Figure S1).

In Australia, influenza is legally notifiable to the Australian National Notifiable Diseases Surveillance System (NNDSS), overseen by the Australian Institute of Health and Welfare. Data on all laboratory confirmed influenza patient notifications were obtained from NNDSS for the study period.

### Study procedures

When conducting prospective recruitment, diagnostic laboratory staff at each hospital informed the study team when GAS was isolated from a normally sterile site in a patient. The study team then approached the patient and their family to invite them to participate. Once written informed consent was obtained, demographic and clinical data were collected and entered onto a RedCap database, including data on clinical outcomes from a follow up survey conducted 6 months post-discharge. When conducting retrospective recruitment, patients were identified in clinical records following a waiver of consent, with relevant data extracted and entered onto the database.

When available, GAS isolates underwent *emm-*gene typing for strain identification using standard laboratory protocols [38].

### Case definitions

All children aged <18 years admitted to a participating hospital during the study period with laboratory confirmed iGAS disease were eligible for inclusion. We defined iGAS disease as the isolation of GAS from a normally sterile bodily site using standard diagnostic microbiological laboratory procedures. ‘Severe disease’ occurred when a patient was admitted to the intensive care unit (ICU), or was treated with (any of) inotropes, haemofiltration, vasopressors, mechanical ventilation or extracorporeal membrane oxygenation (ECMO).

### Statistical analysis

Descriptive epidemiological analyses were performed according to specified demographic patient features, clinical aspects and GAS *emm-*type strain, as reported in the PAEDS database. When calculating iGAS disease and influenza notification incidence rates, census estimate data for the age group and jurisdiction/s of interest were obtained from the Australia Bureau of Statistics website and used as denominator data [39]. Due to the likelihood of missing iGAS patient data due to children not being seen in notifying hospitals, our incidence estimates are described as ‘minimum incidence rates’. Rates were annualised to adjust for data collection occurring over partial years (i.e. 2016 and 2018) using a factor of 2. When calculating annualised rates by year quarter, the adjustment was made using a factor of 4. Where prophylaxis was offered to family or household contact/s of patients, this was recorded in the PAEDS database. Risk ratio (RR) calculations with 95% CI were produced to describe the likelihood of patients’ contacts being offered antibiotics as GAS prophylaxis. The reference group was usually selected on the basis that it included the highest number of patients.

## Results

### Key characteristics of notified patients

A total of 192 patients were notified over the study period, of whom 181 met our criteria for laboratory confirmed iGAS disease and were included. The hospitals that contributed most data on included patients were QCH (43 patients, 23.8% total patients) and RCH (37 patients, 20.4%). Most children, 121 (69.6%) had GAS isolated from blood. IGAS disease was more common in males (107 patients, 59.1%). The majority of patients were less than 5 years old (115, 63.5%), including 32 (17.7%) aged < 1 year old.

Of the 181 patients, 21 (11.6%) identified as having Aboriginal Australian or Torres Strait Islander (ATSI) ethnicity. A total of 74 patients (40.9%) had severe disease and 26 patients (14.4%) had STSS. Although the majority, (122 patients, 67.4%) made a full recovery, 5 children died and the remainder were left with physical deficits and/or ongoing disability at 6 months post-discharge (Table 1).

**Table 1:** Key demographic and clinical characteristics of children with iGAS disease notified to the PAEDS Network, Australia, 1 July 2016–30 June 2018

	Patients (*N* = 181)	
	Number	Proportion of total patients (%)^a^
Age in years		
< 1 year	32	17.7
1–4 years	83	45.9
5–9 years	42	23.2
10–14 years	21	11.6
15–17 years	3	1.7
Sex		
Female	74	40.9
Male	107	59.1
Indigenous Australian ethnicity		
Yes	21	11.6
No	159	87.8
N/A^b^	1	0.6
Country of Birth		
Australia	167	92.3
Other	6	3.3
N/A^b^	8	4.4
Disease severity		
Not severe	107	59.1
Severe	74	40.9
Disease outcome		
Deficit (can improve)	48	26.5
Disability (permanent)	1	0.6
Deficit and disability	4	2.2
Deceased	5	2.8
Full recovery	122	67.4
N/A^b^	1	0.6
Hospital		
QCH	43	23.8
RCH	37	20.4
PCH	27	14.9
MCH	24	13.3
CHW	23	12.7
WCH	22	12.2
RDH	5	2.8

^a^Due to rounding, some totals will not add to 100.0%

^b^N/A: Information not available

*QCH* Queensland Children’s Hospital, Queensland; *RCH* Royal Children’s Hospital Melbourne, Victoria; *PCH* Perth Children’s Hospital, Western Australia; *MCH* Monash Children’s Hospital, Victoria; *CHW* Children’s Hospital at Westmead, New South Wales; *WCH* Women’s and Children’s Hospital, South Australia; *RDH* Royal Darwin Hospital, Northern Territory

Retrospective reviews identified 79 (43.6%) patients, with others recruited prospectively (Additional file 1: Table S1).

Antibiotic prophylaxis was offered to family or other household contacts of 85 patients (47.0%, Table 2).

**Table 2:** Contact prophylaxis offered to household contacts of children with iGAS disease notified to the PAEDS Network, Australia, 1 July 2016–30 June 2018

	Total patients (N)	Patients with contact prophylaxis offered (n)	Proportion of total patients with contact prophylaxis offered (%)^a^	Risk ratio of offering contact prophylaxis (RR, 95% CI)^b^
Total	181	85	47.0	–
Gender				
Female	74	36	48.6	Ref
Male	107	49	45.8	0.94 (0.69–1.29)
Hospital				
RCH	37	30	81.1	Ref
MCH	24	14	58.3	0.72 (0.50–1.04)
QCH	43	2	4.7	**0.06 (0.01–0.22)**
WCH	22	15	68.2	0.84 (0.61–1.16)
RDH	5	0	0.0	*Not calculable*
PCH	27	20	74.1	0.91 (0.70–1.20)
CHW	23	4	17.4	**0.21 (0.09–0.53)**
Disease severity				
Not severe	107	48	44.9	Ref
Severe	74	37	50.0	1.11 (0.82–1.52)
Age group				
< 1 year	32	16	50.0	0.99 (0.66–1.48)
1–4 years	83	42	50.6	Ref
5–9 years	42	19	45.2	0.89 (0.60–1.33)
10–14 years	21	6	28.6	0.56 (0.28–1.15)
15–17 years	3	2	66.7	1.32 (0.57–3.02)
Indigenous Australian ethnicity				
Yes	21	8	38.1	0.79 (0.45–1.40)
No/Don’t know	160	77	48.1	Ref
Patient with a household contacts aged < 10 years				
Yes	116	69	59.5	**2.42 (1.54–3.79)**
No/Don’t know	65	16	24.6	Ref
Severe disease				
Yes	74	37	50.0	1.11 (0.82–1.52)
No	107	48	44.9	Ref

^a^Due to rounding, some totals will add to >100.0%

^b^Bold type indicates result is statistically significant

*RCH* Royal Children’s Hospital Melbourne, Victoria; *MCH* Monash Children’s Hospital, Victoria; *QCH* Queensland Children’s Hospital, Queensland; *WCH* Women’s and Children’s Hospital, South Australia; *RDH* Royal Darwin Hospital, Northern Territory; *PCH* Perth Children’s Hospital, Western Australia; *CHW* Children’s Hospital at Westmead, New South Wales

Of patients with severe disease, 37 of 74 (50.0%) had antibiotic prophylaxis offered to contacts, compared to 48 of 107 patients (44.9%) with non-severe disease. There appeared to be a wide range of practices, with antibiotic prophylaxis offered to 0.0–81.1% of patients by hospital. The likelihood of household contacts being offered secondary prophylaxis was higher when the patient had household contact/s aged <10 years old (RR: 2.42, 95% CI: 1.54–3.79).

### Annualised minimum incidence rate of paediatric iGAS disease

**Fig. 1:**
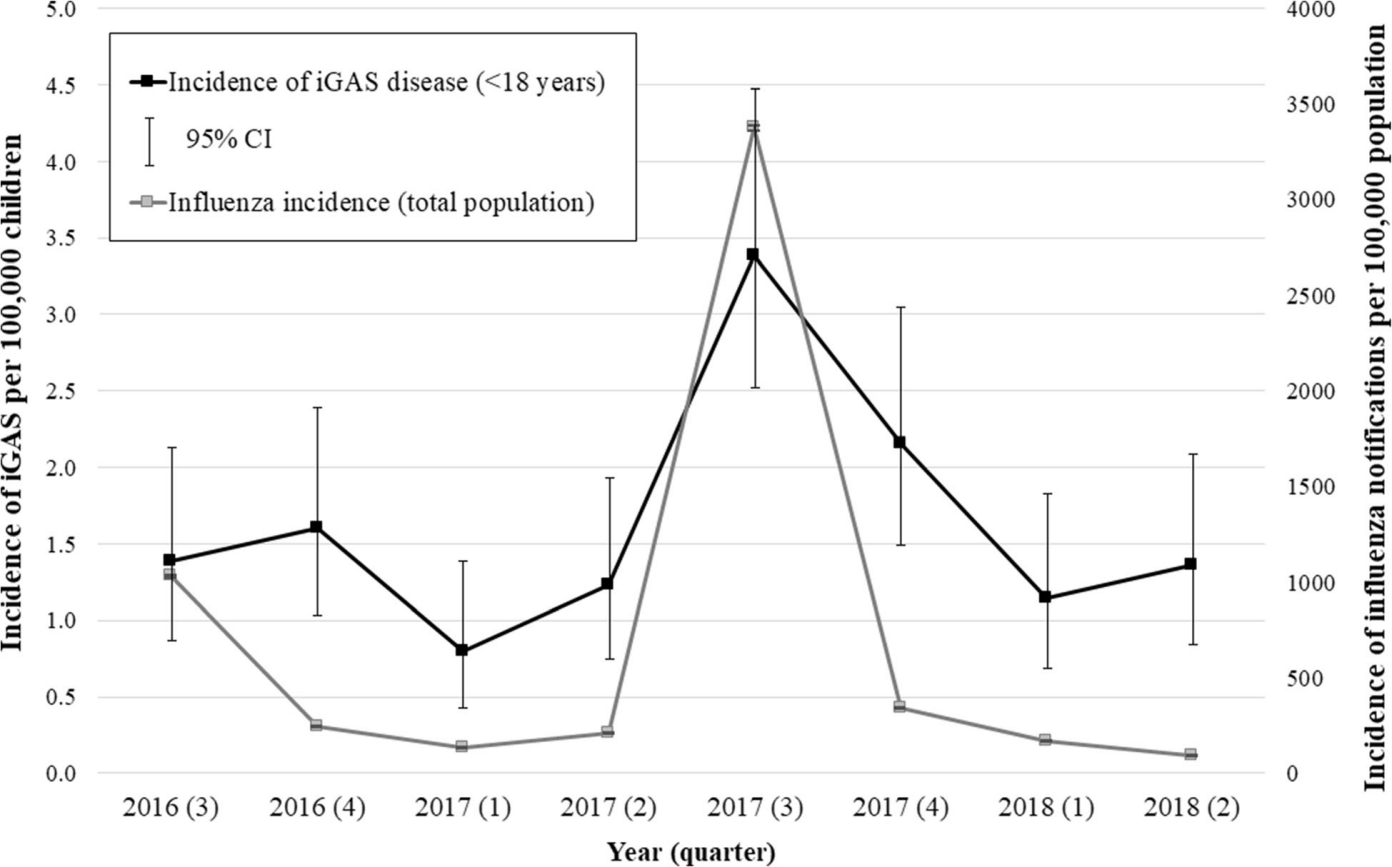
Annualised minimum incidence of notified iGAS (<18 years) and influenza^1^, Australia, July 2016–June 2018. ^**1**^ Annualised incidence of influenza notifications to the Australian Institute of Health and Welfare for the total population

**Fig. 2:**
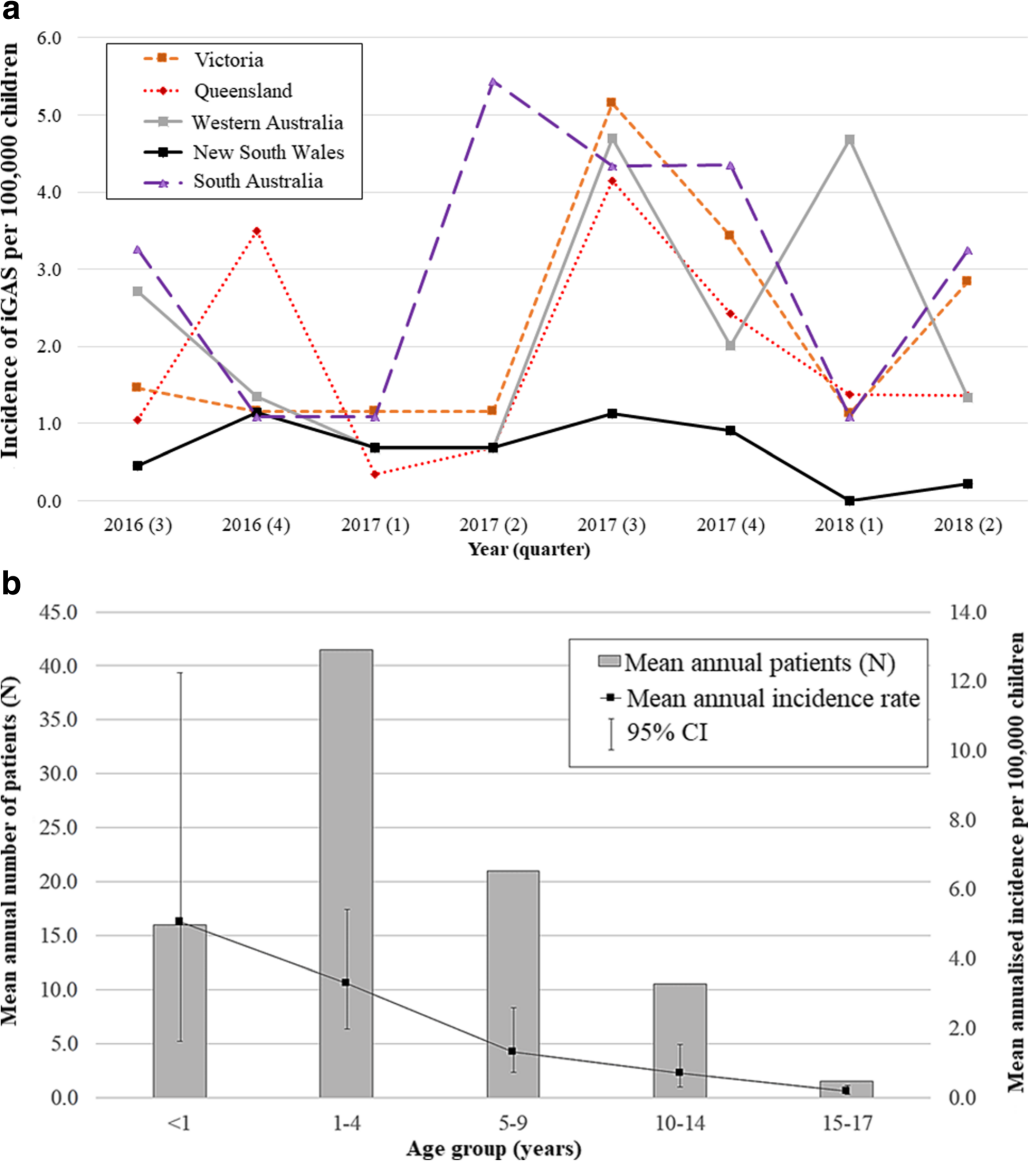
**a** Annualised minimum incidence of notified iGAS (<18 years), Australia, July 2016–June 2018; by jurisdiction. **b** Mean annual patients and minimum iGAS incidence (<18 years), Australia, July 2016–June 2018; age group

The annualised minimum incidence rate of paediatric iGAS disease for all children aged <18 years old is shown in Figs. 1, 2a. Rates are displayed for the whole country and by jurisdiction. The mean minimum incidence rate was 1.6 (95% CI: 1.1–2.3)/100,000 children for the whole study period. This rate was highest during the third quarter of 2017; 3.4 (95% CI: 2.5–4.5)/100,000 children; 47 patients, and lowest during the first quarter of 2017; 0.8 (95% CI: 0.4–1.4)/100,000 children, 11 patients. This pattern followed trends in the national incidence rate of influenza notifications for the total population, with a low burden during the first quarter of 2017; 135.8 notifications (95% CI: 132.9–138.7)/100,000 population, which preceded a sharply increased peak rate in the third quarter of that year; 3376.1 notifications (95% CI: 3391.0-3362.2)/100,000 population, Fig. 1.

The mean annualised minimum incidence rate for ATSI children (<18 years old) across the study period was 6.7 (95% CI: 4.0–11.1)/100,000, 4.1-fold higher than for all children (1.6, 95% CI: 1.1–2.3/100,000).

The NT had the highest mean annualised minimum incidence rate; 4.0, (95% CI: 1.5–7.9)/100,000 children, although with small patient numbers (5 patients in total), followed by SA; 3.0, (95% CI: 1.2–6.0)/100,000, WA; 2.3 (95% CI: 1.1–4.9)/100,000, VIC; 2.2 (95% CI: 1.2–3.8)/100,000, QLD; 1.9 (95% CI: 1.0–3.5)/100,000, and NSW; 0.7 (95% CI: 0.2–1.3)/100,000 (Fig. 2a).

Figure 2b shows the mean annualised minimum incidence rate of paediatric iGAS disease by age group and the mean annual number of patients. The minimum incidence was consistently highest for infants <1 year old; 5.1 (95% CI: 1.6–12.2)/100,000, 32 patients, with rates decreasing for older age groups (Fig. 2b). The minimum incidence for all age groups was highest during the third quarter of 2017, with the exception of 15–17 year old children.

### *emm*-type distribution of patient isolates

**Fig. 3:**
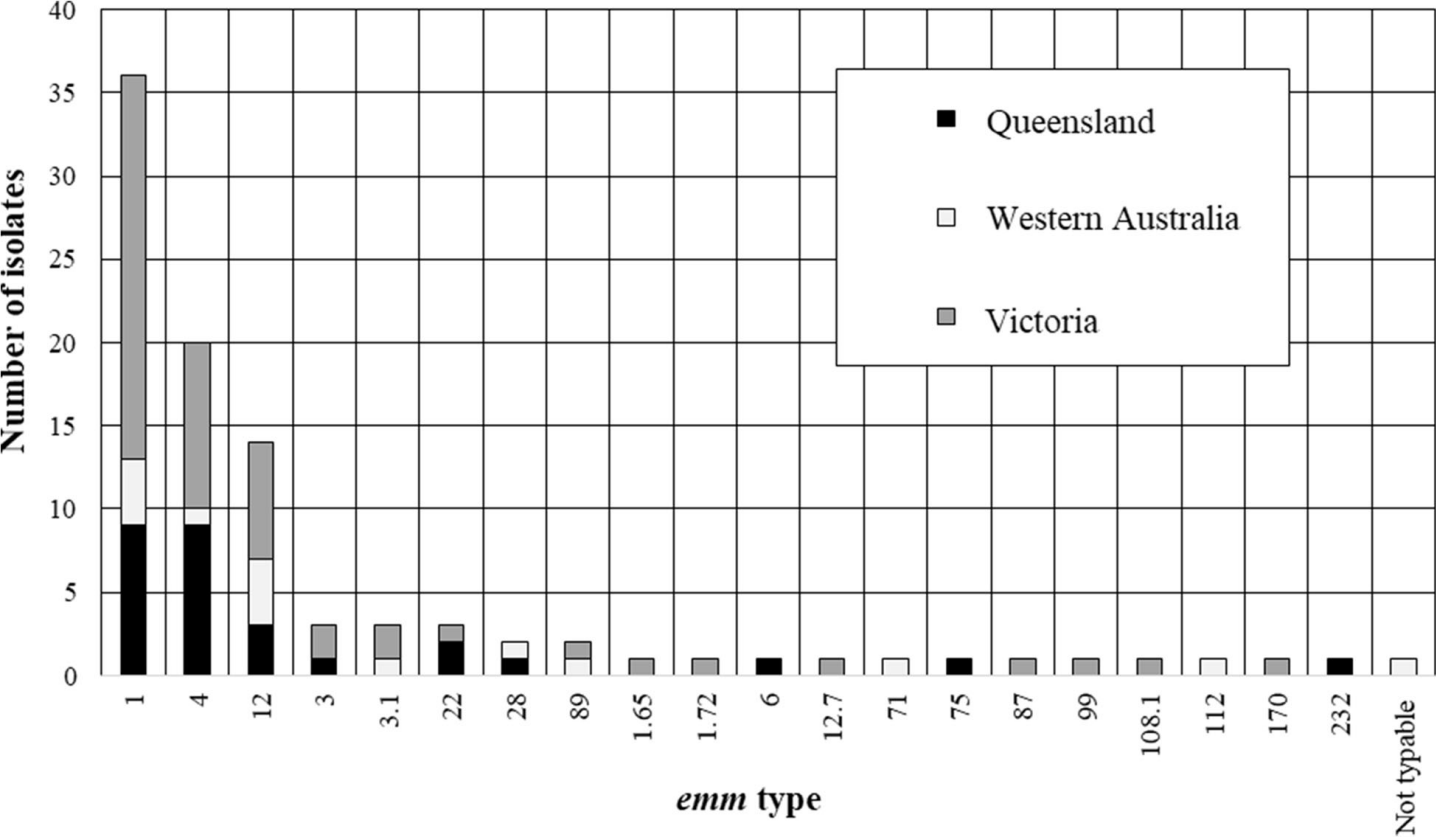
Distribution of iGAS patients (<18 years), Australia, July 2016–June 2018; by streptococcal *emm*-type

There were 96 patients (53.0%) with GAS strain data available (from RCH, MCH, QCH, PCH)*.* A total of 20 different *emm-*types were identified*.* The most common was *emm-1* (36 isolates, 37.5% of those with strain data), followed by *emm-4* (20 isolates, 20.8%) and *emm-12* (14 isolates, 14.6%, Additional file 1: Table S2). Most patients with strain data (53, 55.2%) were hospitalised in Victoria. Fig. 3 shows the distribution of *emm-*types by Australian jurisdiction.

## Discussion

Data collected through the national PAEDS Network has permitted the epidemiological distribution of paediatric iGAS disease and associated *emm*-types to be clearly described over the two-year study period. A concerning burden of disease was documented, concentrated among young children and infants, including a high proportion (12%) of Australian Aboriginal and Torres Strait Islander people, who comprise 3% of the national Australian population. As indicated by previous research in Victoria, Australia, the rate of paediatric iGAS disease among our cohort appeared to broadly correlate with national notifications for influenza, although on a greatly reduced scale [31]. Cases of influenza and iGAS co-infection have been frequently reported in the international literature [40]. Increases in iGAS disease have been correlated with high rates of influenza in several overseas settings, including the UK, Sweden, and Israel [41–43]. While the pathogenic pathways mediating an increased risk of iGAS disease with concurrent/antecedent influenza are not fully understood, certain influenza proteins (such as haemagglutinin) might enhance GAS virulence and/or suppress the immune response [40, 44, 45]. Our findings support the hypothesis that influenza vaccination may offer some protection against iGAS disease, as indicated by a study of US military recruits [44].

We have shown a high morbidity and mortality burden associated with iGAS in Australian children. Our national minimum incidence estimate of 1.6 iGAS patients per 100,000 children is comparable to the rate reported for 5–17 year-old children in Alaska (1.8/100,000 during 2001–2013), [29] and for 5–9 year-old children in the UK during 2017–2018 [21]. Our minimum incidence estimate for children aged < 1 year (5.1/100,000) is close to the reported rate for this age group in Canada (4.8/100,000 during 2001) [15] and in the US (5.3/100,000 during 2000–2004) [5]. Over 40% of the iGAS patients identified by our study were categorised as having severe disease. Furthermore, five children died and nearly 30% were left with a physical deficit and/or disability following hospital discharge. All Australian states that contributed data to the PAEDS Network were affected by paediatric iGAS disease. The national mean annualised minimum incidence rate (1.6/100,000 children) for paediatric iGAS disease is similar to the rate of Meningococcal infection (1.5/100,000 total population in 2017), which is a nationally notifiable condition in Australia [46] and exerts a similar high morbidity and mortality burden to iGAS disease [47]. Further, the risk of secondary iGAS disease among household/family contacts (a 2000-times increased risk) is even higher than the risk of secondary Meningococcal infection (500–800-times increased risk). These findings support the need for iGAS patient notification and evaluation of contact prophylaxis/education for the prevention of secondary disease [7, 8, 48].

Geographic disparities in contact prophylaxis for iGAS patients were apparent. The 2.4-fold increased likelihood of contact prophylaxis being offered when a patient had household contacts aged <10 years old may indicate clinical recognition of the need to protect young children cohabiting with patients from GAS transmission. The geographic variation in offering contact prophylaxis between hospitals, however, illustrates the need for a consensus national recommendation. Despite infants consistently demonstrating the highest rate of iGAS disease, [8] household contacts of infant patients were no more likely to be offered prophylaxis than older patients’ household contacts. Any national recommendation should also take into consideration the especially pronounced risk of infection in mother-neonate pairs [8].

Twenty different *emm*-types were associated with iGAS disease, with the three most prevalent strains (*emm*-1, −4, and − 12) accounting for nearly three-quarters of patients (for whom strain data was available). This strain diversity is much less than observed among the general population of Victoria or of Sydney, NSW [49, 50]. A GAS vaccine would need to possess broad strain coverage in order to effectively prevent invasive disease - but should a vaccine incorporate the three most prevalent strains observed in this study, it would potentially prevent nearly three-quarters of disease in paediatric patients.

Under-notification of patients is likely to be a serious issue affecting the completeness and ultimately, the usefulness, of surveillance data, including that used in this study. Reporting to the PAEDS Network is voluntary and is limited to active surveillance at seven sentinel tertiary hospitals, so smaller regional hospitals and other tertiary hospitals will have managed iGAS disease patients without contributing data. Such under-notification will have impacted on the rate estimations and may particularly affect remote areas of Australia, including areas with significant ATSI populations. Consequently, the true extent of ATSI children’s overrepresentation in iGAS disease rates may be higher than was shown by this analysis. In addition, iGAS disease patients with more severe symptoms may require transfer to a (notifying) hospital for specialist care. Thus less severe paediatric iGAS disease patients may have been missed. Furthermore, a requirement of case notification was the identification of GAS from a normally sterile body site. Severe cases excluded from our analyses due to this requirement are likely to be very few in number, however STSS and necrotizing fasciitis can occur without GAS entering a sterile site [3, 51]. The extent to which the true rate is underestimated is unknown and will vary by jurisdiction. For completeness, national notifications should include all iGAS cases regardless of whether a GAS isolate was obtained from a normally sterile site. Other limitations include an absence of information on secondary prophylaxis compliance and incomplete strain data.

## Conclusions

Various means of categorising ‘severe’ iGAS diseases have been used in the literature [52, 53], however on the basis of the frequency of iGAS disease identified here and the high disease burden, we advocate for urgent public health action to improve surveillance and optimise prevention activities. There is a clear need for a consensus national recommendation around the use of contact prophylaxis. The lack of mandatory patient notification limits the ability of public health programmes to effectively target, prevent and control this condition. Making iGAS disease notifiable at the national level would help to inform public health and research initiatives aiming to reduce the impact of this condition [49]. The momentum for GAS vaccine development is supported by increasing the awareness of iGAS disease morbidity and mortality, both nationally and internationally.

## Supplementary information

**Additional file 1: Table S1.** Recruitment method by hospital, notified iGAS patients (<18 years), Australia, July 2016–June 2018. **Table S2.** emm-types (*N* = 96) identified among notified iGAS disease patients (<18 years), Australia, July 2016–June 2018.

**Additional file 2: Figure S1.** Prospective surveillance implementation periods across the seven notifying PAEDS Network sites^1^**.** ^1^Sites are: RDH: Royal Darwin Hospital, Northern Territory; PCH: Perth Children’s Hospital, Western Australia; WCH: Women’s and Children’s Hospital, South Australia; CHW: Children’s Hospital at Westmead, New South Wales; QCH: Queensland Children’s Hospital, Queensland; RCH: Royal Children’s Hospital Melbourne, Victoria; MCH: Monash Children’s Hospital, Victoria).

**Additional file 3: Figure S2.** Location of the seven notifying PAEDS Network sites and major cities, Australia.

## Data Availability

Due to the potentially identifiable nature of the raw data, these are not publicly available and will not be shared.

## Abbreviations

ATSI: Aboriginal Australian or Torres Strait Islander
CHW: Children’s Hospital at Westmead, New South Wales
CI: Confidence interval
CRF: Case fatality rate
ECMO: Extracorporeal membrane oxygenation
ICU: Intensive care unit
iGAS: Invasive group A Streptococcus
MCH: Monash Children’s Hospital, Victoria
NNDSS: Australian National Notifiable Diseases Surveillance System
PAEDS: Paediatric Active Enhanced Disease Surveillance
PCH: Perth Children’s Hospital, Western Australia
QCH: Queensland Children’s Hospital, Queensland
RCH: Royal Children’s Hospital Melbourne, Victoria
RDH: Royal Darwin Hospital, Northern Territory
RR: Risk Ratio
STSS: Streptococcal toxic shock syndrome
US: United States of America
WCH: Women’s and Children’s Hospital, South Australia
WHO: World Health Organization
