# Correction to: Invasive group A Streptococcus disease in Australian children: 2016 to 2018 – a descriptive cohort study

**DOI:** 10.1186/s12889-021-10798-6

**Published:** 2021-05-03

**Authors:** Jane Oliver, Elise Thielemans, Alissa McMinn, Ciara Baker, Philip N. Britton, Julia E. Clark, Helen S. Marshall, Christopher C. Blyth, Joshua Francis, Jim Buttery, Andrew C. Steer, Nigel W. Crawford, on behalf of the PAEDS investigators

**Affiliations:** 1 Murdoch Children’s Research Institute, Royal Children’s Hospital, Flemington Rd, Parkville, Victoria 3052 Australia; 2 The Peter Doherty Institute for Infection and Immunity, University of Melbourne, Melbourne, Victoria Australia; 3 Université Libre de Bruxelles, Bruxelles, Belgium; 4 The Children’s Hospital at Westmead, Sydney, Australia; 5 Medical School University of Sydney, Sydney, New South Wales Australia; 6 Queensland Children’s Hospital, and School of Clinical Medicine, University of Queensland, Brisbane, Queensland Australia; 7 Women’s and Children’s Hospital, Adelaide, South Australia Australia; 8 School of Medicine angeid Telethon Kids Institute, University of Western Australia, Perth, Australia; 9 Perth Children’s Hospital, Perth, Western Australia Australia; 10 PathWest Laboratory Medicine, Nedlands, Perth, Australia; 11 Royal Darwin Hospital, Darwin, Northern Territory, Australia; 12 Menzies School of Health Research, Darwin, Northern Territory, Australia; 13 Monash Health, Monash University, Melbourne, Victoria Australia

**Correction to: BMC Public Health 19, 1750 (2019)**

**https://doi.org/10.1186/s12889-019-8085-2**

It was highlighted that the original article [1] contained an error in rates in the section “Annualised minimum incidence rate of paediatric iGAS disease”. This Correction article shows the incorrect and correct sentence. The authors would like to apologize to the readers for the inconvenience. The original article has been updated.

**Incorrect**

The mean annualised minimum incidence rate for ATSI children (<18 years old) across the study period

was 6.7 (95% CI: 4.0–11.1)/100,000, 4.1-fold higher than for all children (1.6, 95% CI: 1.1–2.3/100,000).

**Correct**

The mean annualised minimum incidence rate for ATSI children (<18 years old) across the study period

was 3.4 (95% CI: 2.1–5.7)/100,000, 2.1-fold higher than for all children (1.6, 95% CI: 1.1–2.3/100,000).
