# Supplementary material for: Invasive group A Streptococcus disease in Australian children: 2016 to 2018 – a descriptive cohort study
Source: BMC Public Health. 2019 Dec 30;19:1750. doi: 10.1186/s12889-019-8085-2 (PMC6937995; doi:10.1186/s12889-019-8085-2)
Supplement: Supplementary file 1 — Additional file 1: Table S1. Recruitment method by hospital, notified iGAS patients (<18 years), Australia, July 2016–June 2018. Table S2. emm-types (N = 96) identified among notified iGAS disease patients (<18 years), Australia, July 2016–June 2018. [file 12889_2019_8085_MOESM1_ESM.docx]

# **Table S1**. Recruitment method by hospital, notified iGAS patients (<18 years), Australia, July 2016-June 2018

| **Recruitment method** | **RCH** | **MCH** | **QCH** | **WCH** | **RDH** | **PCH** | **CHW** | **Total** |
| --- | --- | --- | --- | --- | --- | --- | --- | --- |
| Prospective  n (%) | 10 (27.0) | 8  (33.3) | 25 (58.1) | 9  (40.9) | 5 (100.0) | 15 (55.6) | 7  (30.4) | 79 (43.6) |
| Retrospective  n (%) | 27 (73.0) | 16 (66.7) | 18 (41.9) | 13 (59.1) | 0  (0.0) | 12 (44.4) | 16  (69.6) | 102 (56.4) |
| Total n | 37 | 24 | 43 | 22 | 5 | 27 | 23 | **181** |

**Table S2.** *emm*-types (N=96) identified among notified iGAS disease patients (<18 years), Australia, July 2016–June 2018

| ***emm-type*** | **Specimens (N)** | **Proportion of specimens with *emm-type* data available (%)** | **Cumulative proportion of specimens with *emm-type* data available (%)^1^** |
| --- | --- | --- | --- |
| 1 | 36 | 37.5 | 37.5 |
| 4 | 20 | 20.8 | 58.3 |
| 12 | 14 | 14.6 | 72.9 |
| 3 | 3 | 3.1 | 76.0 |
| 3.1 | 3 | 3.1 | 79.1 |
| 22 | 3 | 3.1 | 82.2 |
| 28 | 2 | 2.1 | 84.4 |
| 89 | 2 | 2.1 | 86.6 |
| 1.65 | 1 | 1.0 | 87.6 |
| 1.72 | 1 | 1.0 | 88.6 |
| 6 | 1 | 1.0 | 89.6 |
| 12.7 | 1 | 1.0 | 90.6 |
| 71 | 1 | 1.0 | 91.6 |
| 75 | 1 | 1.0 | 92.6 |
| 87 | 1 | 1.0 | 93.6 |
| 99 | 1 | 1.0 | 94.6 |
| 108.1 | 1 | 1.0 | 95.6 |
| 112 | 1 | 1.0 | 96.6 |
| 170 | 1 | 1.0 | 97.6 |
| 232 | 1 | 1.0 | 98.6 |
| Not typable | 1 | 1.0 | 99.6 |
| No *emm-*type data available | 85 | - | - |

^1^ Cumulative proportion is added from top down, eg. 58.3 in the *emm-*4 row refers to the sum proportion of isolates that were emm-1 (37.5%) and emm 4 (20.8%). Rounding to 1 decimal place results in a cumulative total of <100.0% reported.
