# Supplementary figures and images for: Invasive group A Streptococcus disease in Australian children: 2016 to 2018 – a descriptive cohort study
Source: BMC Public Health. 2019 Dec 30;19:1750. doi: 10.1186/s12889-019-8085-2 (PMC6937995; doi:10.1186/s12889-019-8085-2)

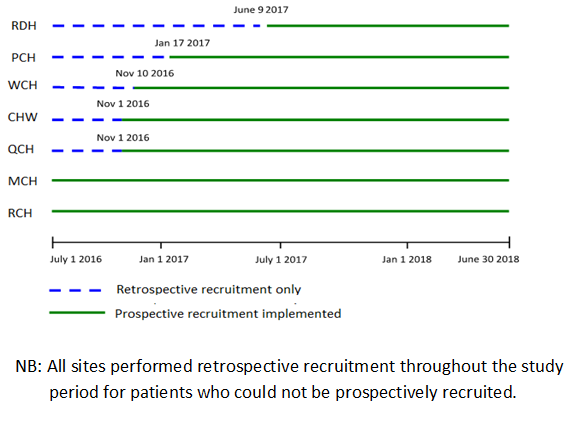

Supplement: Supplementary file 2 — Additional file 2: Figure S1. Prospective surveillance implementation periods across the seven notifying PAEDS Network sites1. 1Sites are: RDH: Royal Darwin Hospital, Northern Territory; PCH: Perth Children’s Hospital, Western Australia; WCH: Women’s and Children’s Hospital, South Australia; CHW: Children’s Hospital at Westmead, New South Wales; QCH: Queensland Children’s Hospital, Queensland; RCH: Royal Children’s Hospital Melbourne, Victoria; MCH: Monash Children’s Hospital, Victoria). [file 12889_2019_8085_MOESM2_ESM.tif]

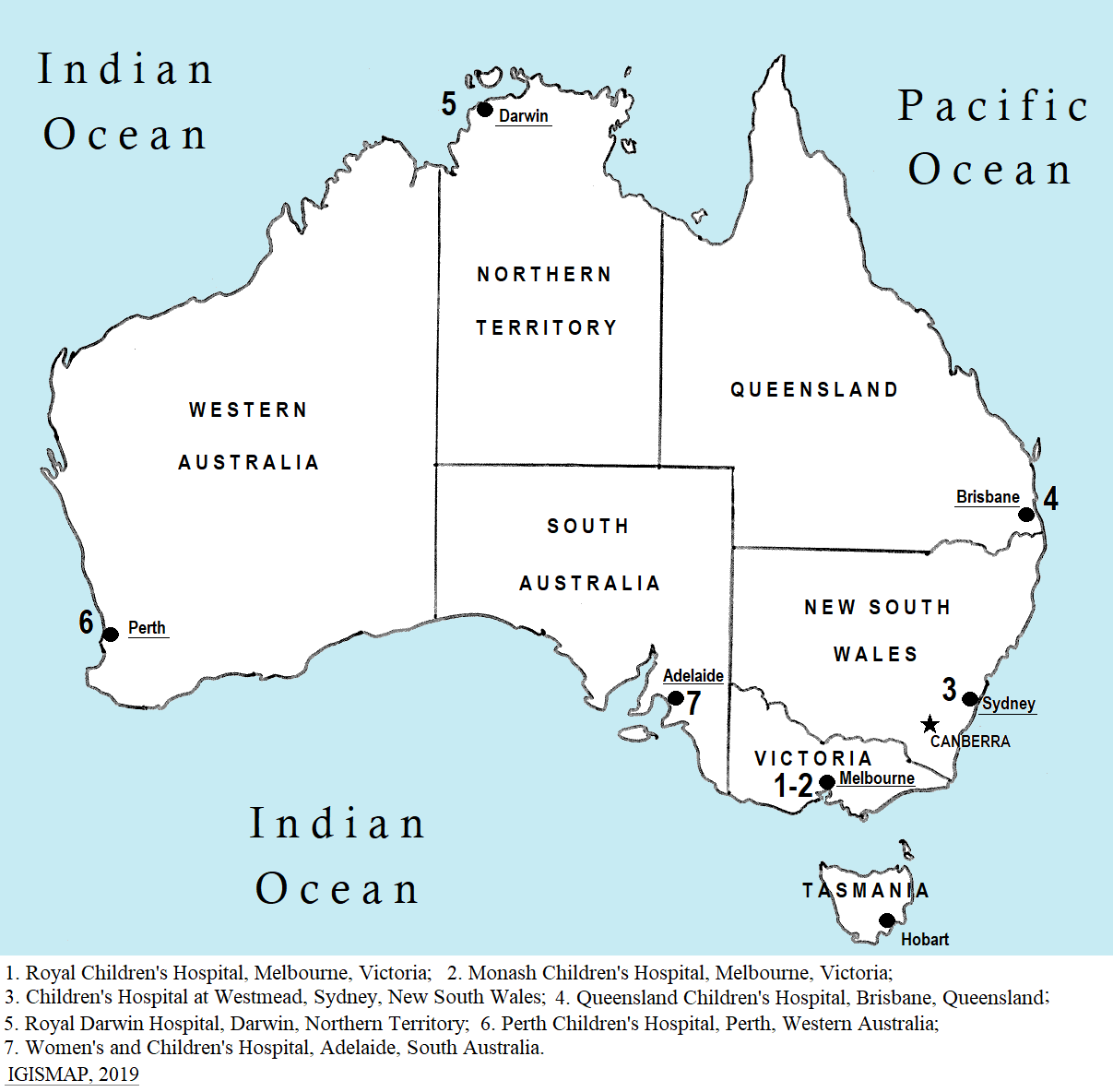

Supplement: Supplementary file 3 — Additional file 3: Figure S2. Location of the seven notifying PAEDS Network sites and major cities, Australia. [file 12889_2019_8085_MOESM3_ESM.tif]
